# Contribution of Somatic Ras/Raf/Mitogen-Activated Protein Kinase Variants in the Hippocampus in Drug-Resistant Mesial Temporal Lobe Epilepsy

**DOI:** 10.1001/jamaneurol.2023.0473

**Published:** 2023-05-01

**Authors:** Sattar Khoshkhoo, Yilan Wang, Yasmine Chahine, E. Zeynep Erson-Omay, Stephanie M. Robert, Emre Kiziltug, Eyiyemisi C. Damisah, Carol Nelson-Williams, Guangya Zhu, Wenna Kong, August Yue Huang, Edward Stronge, H. Westley Phillips, Brian H. Chhouk, Sara Bizzotto, Ming Hui Chen, Thiuni N. Adikari, Zimeng Ye, Tom Witkowski, Dulcie Lai, Nadine Lee, Julie Lokan, Ingrid E. Scheffer, Samuel F. Berkovic, Shozeb Haider, Michael S. Hildebrand, Edward Yang, Murat Gunel, Richard P. Lifton, R. Mark Richardson, Ingmar Blümcke, Sanda Alexandrescu, Anita Huttner, Erin L. Heinzen, Jidong Zhu, Annapurna Poduri, Nihal DeLanerolle, Dennis D. Spencer, Eunjung Alice Lee, Christopher A. Walsh, Kristopher T. Kahle

**Affiliations:** 1 Department of Neurology, Brigham and Women’s Hospital, Harvard Medical School, Boston, Massachusetts; 2 Division of Genetics and Genomics, Manton Center for Orphan Disease Research, Boston Children’s Hospital, Boston, Massachusetts; 3 Broad Institute of MIT and Harvard, Cambridge, Massachusetts; 4 Program in Biological and Biomedical Sciences, Harvard Medical School, Boston, Massachusetts; 5 Department of Neurosurgery, Yale University School of Medicine, New Haven, Connecticut; 6 Department of Genetics, Yale University School of Medicine, New Haven, Connecticut; 7 Interdisciplinary Research Center on Biology and Chemistry, Shanghai Institute of Organic Chemistry, Chinese Academy of Sciences, Shanghai, China; 8 Department of Pediatrics, Harvard Medical School, Boston, Massachusetts; 9 Harvard Medical School, Boston, Massachusetts; 10 Department of Neurosurgery, David Geffen School of Medicine, University of California, Los Angeles; 11 Sorbonne University, Paris Brain Institute (ICM), National Institute of Health and Medical Research (INSERM), National Center for Scientific Research (CNRS), Paris, France; 12 Department of Medicine (Austin Health), University of Melbourne, Heidelberg, Australia; 13 Division of Pharmacotherapy and Experimental Therapeutics, Eshelman School of Pharmacy, University of North Carolina at Chapel Hill; 14 Department of Anatomical Pathology, Austin Health, Heidelberg, Australia; 15 Murdoch Children’s Research Institute, Parkville, Australia; 16 Florey Institute of Neuroscience and Mental Health, Heidelberg, Australia; 17 Department of Pediatrics, University of Melbourne, Royal Children’s Hospital, Parkville, Australia; 18 Bladin-Berkovic Comprehensive Epilepsy Program, Department of Neurology, Austin Health, Heidelberg, Australia; 19 Department of Pharmaceutical and Biological Chemistry, University College London School of Pharmacy, London, United Kingdom; 20 Department of Radiology, Boston Children’s Hospital, Harvard Medical School, Boston, Massachusetts; 21 Laboratory of Human Genetics and Genomics, The Rockefeller University, New York, New York; 22 Department of Neurosurgery, Massachusetts General Hospital, Boston; 23 Department of Neuropathology, University Hospitals Erlangen, Erlangen, Germany; 24 Epilepsy Center, Cleveland Clinic, Cleveland, Ohio; 25 Department of Pathology, Boston Children’s Hospital, Harvard Medical School, Boston, Massachusetts; 26 Department of Pathology, Yale University School of Medicine, New Haven, Connecticut; 27 Department of Genetics, School of Medicine, University of North Carolina at Chapel Hill; 28 Epilepsy Genetics Program, Division of Epilepsy and Neurophysiology, Department of Neurology, Boston Children’s Hospital, Harvard Medical School, Boston, Massachusetts; 29 Department of Neurology and Pediatrics, Harvard Medical School, Boston, Massachusetts; 30 Allen Discovery Center for Human Brain Evolution, Boston Children’s Hospital, Harvard Medical School, Boston, Massachusetts; 31 Howard Hughes Medical Institute, Boston, Massachusetts; 32 Department of Neurosurgery, Boston Children’s Hospital, Boston, Massachusetts

## Abstract

**Importance:**

Mesial temporal lobe epilepsy (MTLE) is the most common focal epilepsy subtype and is often refractory to antiseizure medications. While most patients with MTLE do not have pathogenic germline genetic variants, the contribution of postzygotic (ie, somatic) variants in the brain is unknown.

**Objective:**

To test the association between pathogenic somatic variants in the hippocampus and MTLE.

**Design, Setting, and Participants:**

This case-control genetic association study analyzed the DNA derived from hippocampal tissue of neurosurgically treated patients with MTLE and age-matched and sex-matched neurotypical controls. Participants treated at level 4 epilepsy centers were enrolled from 1988 through 2019, and clinical data were collected retrospectively. Whole-exome and gene-panel sequencing (each genomic region sequenced more than 500 times on average) were used to identify candidate pathogenic somatic variants. A subset of novel variants was functionally evaluated using cellular and molecular assays. Patients with nonlesional and lesional (mesial temporal sclerosis, focal cortical dysplasia, and low-grade epilepsy–associated tumors) drug-resistant MTLE who underwent anterior medial temporal lobectomy were eligible. All patients with available frozen tissue and appropriate consents were included. Control brain tissue was obtained from neurotypical donors at brain banks. Data were analyzed from June 2020 to August 2022.

**Exposures:**

Drug-resistant MTLE.

**Main Outcomes and Measures:**

Presence and abundance of pathogenic somatic variants in the hippocampus vs the unaffected temporal neocortex.

**Results:**

Of 105 included patients with MTLE, 53 (50.5%) were female, and the median (IQR) age was 32 (26-44) years; of 30 neurotypical controls, 11 (36.7%) were female, and the median (IQR) age was 37 (18-53) years. Eleven pathogenic somatic variants enriched in the hippocampus relative to the unaffected temporal neocortex (median [IQR] variant allele frequency, 1.92 [1.5-2.7] vs 0.3 [0-0.9]; *P* = .01) were detected in patients with MTLE but not in controls. Ten of these variants were in *PTPN11*, *SOS1*, *KRAS*, *BRAF*, and *NF1*, all predicted to constitutively activate Ras/Raf/mitogen-activated protein kinase (MAPK) signaling. Immunohistochemical studies of variant-positive hippocampal tissue demonstrated increased Erk1/2 phosphorylation, indicative of Ras/Raf/MAPK activation, predominantly in glial cells. Molecular assays showed abnormal liquid-liquid phase separation for the *PTPN11* variants as a possible dominant gain-of-function mechanism.

**Conclusions and Relevance:**

Hippocampal somatic variants, particularly those activating Ras/Raf/MAPK signaling, may contribute to the pathogenesis of sporadic, drug-resistant MTLE. These findings may provide a novel genetic mechanism and highlight new therapeutic targets for this common indication for epilepsy surgery.

## Key Points

**Question:**

Do postzygotic (ie, somatic) variants in the hippocampus contribute to mesial temporal lobe epilepsy (MTLE) risk?

**Findings:**

In this genetic association study of 105 neurosurgically treated patients with MTLE and 30 neurotypical brain donors, 11 pathogenic somatic variants enriched in the hippocampus were detected in unrelated patients with MTLE but not in the controls. Ten of these variants were in *PTPN11*, *SOS1*, *KRAS*, *BRAF*, and *NF1*, all predicted to constitutively activate Ras/Raf/mitogen-activated protein kinase (MAPK) signaling.

**Meaning:**

In this study, hippocampal somatic variants, in particular those activating Ras/Raf/MAPK signaling, may contribute to sporadic, drug-resistant MTLE.

## Teaser

This genetic association study evaluates the association between pathogenic somatic variants in the hippocampus and mesial temporal lobe epilepsy.

## Introduction

Epilepsy is a debilitating chronic neurologic condition that affects 1 in 26 people (3% to 4% lifetime risk).^1^ The most common focal epilepsy subtype, mesial temporal lobe epilepsy (MTLE), is often resistant to antiseizure medications and requires neurosurgical intervention (eg, anterior temporal lobectomy) in roughly one-third of patients, with attendant morbidity.^2^ While MTLE has been associated with initial precipitating insults, such as prolonged febrile seizures^3^ and trauma,^4^ its etiology is debated and its pathophysiology is poorly understood. With a few exceptions,^5,6,7,8^ whole-exome sequencing (WES) and gene-panel sequencing (GPS) studies of blood-derived and buccal-derived DNA have had minimal success in identifying genetic determinants of focal epilepsies, such as MTLE,^9,10,11,12,13,14^ which typically occur in individuals without a family history of the disease.

Recently, postzygotic (ie, somatic) variants have emerged as a major cause of focal epilepsies associated with focal cortical dysplasia (FCD).^15^ Pathogenic somatic variants in FCD are present in only a fraction of cells (1% to 10% typically), creating a mosaic with admixed variant (variant-positive) and nonvariant cells. The level of mosaicism, as defined by variant allele frequency (VAF), in focal epilepsy usually correlates with the size and brain regional distribution of the lesion.^15^ Most somatic variants identified in FCD are seen in samples with type 2B histopathological classification and result in activation of PI3K/Akt/mammalian target of rapamycin (mTOR) pathway genes that typically produce a lesion visible on magnetic resonance imaging.^15^ However, somatic variants in non–PI3K/Akt/mTOR genes can also cause focal epilepsies, without always producing a radiographically evident dysplasia, even at high VAFs.^16^

In this study, we tested the hypothesis that somatic variants enriched in the hippocampus are an important pathogenic mechanism underlying drug-resistant MTLE. We used deep WES and GPS to identify somatic variants in the hippocampal tissue from neurosurgically treated patients with MTLE and neurotypical controls.

## Methods

### Oversight and Study Procedures

The main study group included patients with MTLE at Yale-New Haven Hospital, New Haven, Connecticut; Boston Children’s Hospital, Boston, Massachusetts: and Austin Hospital, Heidelberg, Australia. Written informed consent was obtained from all patients or their guardians with local institutional review board or ethics committee approval. All research performed with samples obtained from patients was approved by the Institutional Review Board at Boston Children’s Hospital.

### Study Participants

This case-control study was designed to investigate the association between somatic genetic variants and MTLE. Patients with nonlesional and lesional (mesial temporal sclerosis [MTS], FCD, and low-grade epilepsy–associated tumors [LEATs], including ganglioglioma, dysembryoplastic neuroepithelial tumor, and multinodular and vacuolating neuronal tumor) drug-resistant MTLE who underwent anterior mesial temporal lobe resection were included. All non-LEAT primary brain tumors were excluded. Neurotypical control brain tissue was obtained from the National Institutes of Health NeuroBioBank at the University of Maryland, Baltimore, and the University of Miami, Miami, Florida, as well as the European Epilepsy Brain Bank, Erlangen, Germany.

### Target Capture, Sequencing, and Somatic Variant Analysis

WES and GPS (each genomic region sequenced more than 500 times on average) was performed on DNA extracted from fresh-frozen brain tissue or nonbrain tissue to detect somatic variants, which were independently validated with amplicon sequencing or droplet digital polymerase chain reaction.

### Cellular and Molecular Studies

Cell lines were transfected with wild-type and variant human gene constructs and underwent immunoblotting and liquid-liquid phase separation (LLPS) assays. Additional histopathology experiments were performed on archival paraffin-embedded tissue as needed.

### Statistical Analysis

Pairwise comparisons were made with Fisher exact test, *t* test, Wilcoxon rank-sum and signed rank tests, and the binomial test; 2-tailed *P* values less than .05 were considered statistically significant. Pathway enrichment analysis^17^ and gene set overrepresentation analysis were evaluated using the hypergeometric test; false discovery rate–adjusted *P* values less than .05 were considered statistically significant. Python version 3.7.4 (Python Software Foundation) was used for all statistical analysis. Additional details are provided in eMethods in Supplement 1.

## Results

### Patients

Surgical samples were obtained from 105 patients who underwent anterior medial temporal lobectomy for medication-refractory MTLE from 1988 through 2019. Of 105 included patients with MTLE, 53 (50.5%) were female, and the median (IQR) age was 32 (26-44) years; of 30 neurotypical controls, 11 (36.7%) were female, and the median (IQR) age was 37 (18-53) years. Clinical histopathologic findings included MTS only (n = 91), MTS with LEATs (n = 4), MTS with FCD (n = 2), and non-MTS (n = 8). Detailed clinical and demographic information is provided in eTables 1 and 2 in Supplement 1 and eTable 1 in Supplement 2. All patients had hippocampal tissue available. A subset also had paired temporal neocortical tissue (n = 89) and blood (n = 27) collected. Only fresh-frozen specimens were used for WES or GPS and validation.

### Pathogenic Somatic Variant Discovery and Brain Regional Enrichment Analysis

Hippocampus-derived DNA from patients was sequenced via WES or GPS, with each genomic region sequenced more than 500 times on average (eFigure 1 in Supplement 1), followed by somatic variant calling. After careful bioinformatic measures to improve the accuracy of the somatic variant call set, all the variants previously reported as pathogenic or likely pathogenic in ClinVar^18^ were experimentally tested with amplicon sequencing and/or droplet digital polymerase chain reaction. Of 21 candidate variants tested, 11 were validated. All validated pathogenic or likely pathogenic somatic variants detected in MTLE samples, and their corresponding VAFs, are shown in the Table. Among the tissue samples with pathogenic somatic variants, 5 cases had MTS with LEATs or FCD pathology, with 2.5% to 23.6% mean VAFs. The remaining 6 cases with MTS-only pathology had significantly lower mean VAFs, ranging from 0.8% to 3.3%. We did not identify pathogenic somatic variants in the small non-MTS group.

**Table.** Pathogenic Somatic Variants in Mesial Temporal Lobe Epilepsy

Patient No./sex/age at surgery, y	Clinical diagnosis	Gene	Variant coordinates	Protein change	VAF, %	
					Sequencing (validation) in the hippocampus	Validation in the temporal neocortex
1/Male/30s	MTS	*PTPN11*	NM_002834.5: c.1507G>A	p.G503R	2.4 (1.4-1.5)	<0.2
2/Male/30s	MTS with LEAT	*PTPN11*	NM_002834.4: c.1508G>T	p.G503V	2.9 (2.1-2.5)	<0.2
3/Male/20s	MTS	*PTPN11*	NM_002834.5: c.417G>C	p.E139N	3.3 (3.3)	0.8-0.9
4/Male/20s	MTS	*NF1*	NM_001042492.3: c.654 + 1G>A; germline NM_001042492.3: c.499_502del	Altered splicing p.C167fs	2.2 (2.0)	0.9
5/Female/20s	MTS with LEAT	*NF1*	NM_001042492.3: c.6852-6855del; germline NM_001042492.3: c.6904C>T	p.Y2285fs p.Q2302*	6.3 (6.3)	NA
6/Male/40s	MTS	*KRAS*	NM_004985.5: c.35G>A	p.G12D	1.2 (0.9-0.9)	<0.2
7/Male/<10	MTS with FCD1A	*KRAS*	NM_004985.5: c.35G>T^a^	p.G12V	19.6 (18)	NA
8/Female/50s	MTS	*SOS1*	NM_005633.4: c.810-813del	p.M269del	1.2 (0.52-0.88)	0.6
9/Female/10s	MTS with LEAT	*BRAF*	NM_004333.6: c.1799T>A	p.V600E	7.9 (7.82-7.9)	NA
10/Male/<10	MTS with FCD2A	*BRAF*	NM_004333.6: c.1797A>G	p.K601E	31.3 (17.8)	8.8
11/Female/50s	MTS	*SF3B1*	NM_012433.3: c.2098A>G	p.K700E	1.7 (1.5-1.7)	<0.2

Abbreviations: FCD, focal cortical dysplasia; LEAT, low-grade epilepsy–associated tumor; MTS, mesial temporal sclerosis; NA, not available; VAF, variant allele frequency.

^a^ Has been previously reported in a separate cohort consisting of malformations of cortical development.^23^

Since low VAF somatic variants may have been acquired at later developmental stages and be restricted to a small brain region, we also investigated the presence of pathogenic somatic variants in the unaffected temporal neocortex when paired tissue was available. For all the variants that could be tested in both brain regions (n = 8), the pathogenic variants were undetectable (less than 0.2% VAF in 4 patients) or less common (4 patients) in the temporal neocortex (Table), suggesting that variants were selectively enriched in affected hippocampal tissue (median [IQR] VAF, 1.92 [1.5-2.7] vs 0.3 [0-0.9]; *P* = .01).

Notably, the *PTPN11* (c.1507G>A and c.1508G>T), *KRAS (*c.35G>A and c.35G>T), and *BRAF* (c.1797A>G and c.1799T>A) variants are all located in mutational hot spots for cancer and neurodevelopmental disorders.^19,20,21^ However, except for *KRAS* c.35G>T and *BRAF* c.1799T>A, which have been reported in FCDs^22,23^ and LEATs,^24^ to our knowledge, none of the other somatic variants have been previously described in focal epilepsies. Both patients with *NF1* somatic variants carried a diagnosis of neurofibromatosis type 1 and had known germline variants in the *NF1* gene (c.499_502del and c.6904C>T) based on clinical testing. Our findings of coexistent germline and somatic variants in patients with MTLE are consistent with the established double-hit mechanism in *NF1*-associated pathology.^25^

To test whether pathogenic somatic variants exist in the hippocampus of neurotypical individuals, we performed WES on a cohort of 30 control hippocampal samples (eFigure 1 in Supplement 1). Since normal hippocampal tissue is rarely surgically resected, we used postmortem hippocampal dissections from individuals with no reported neurologic disease. In contrast to the MTLE cohort, we did not identify any pathogenic or likely pathogenic variants in the control samples.

### Clinical Findings in Patients With Activating Ras/Raf/MAPK Somatic Variants

**Figure 1.**
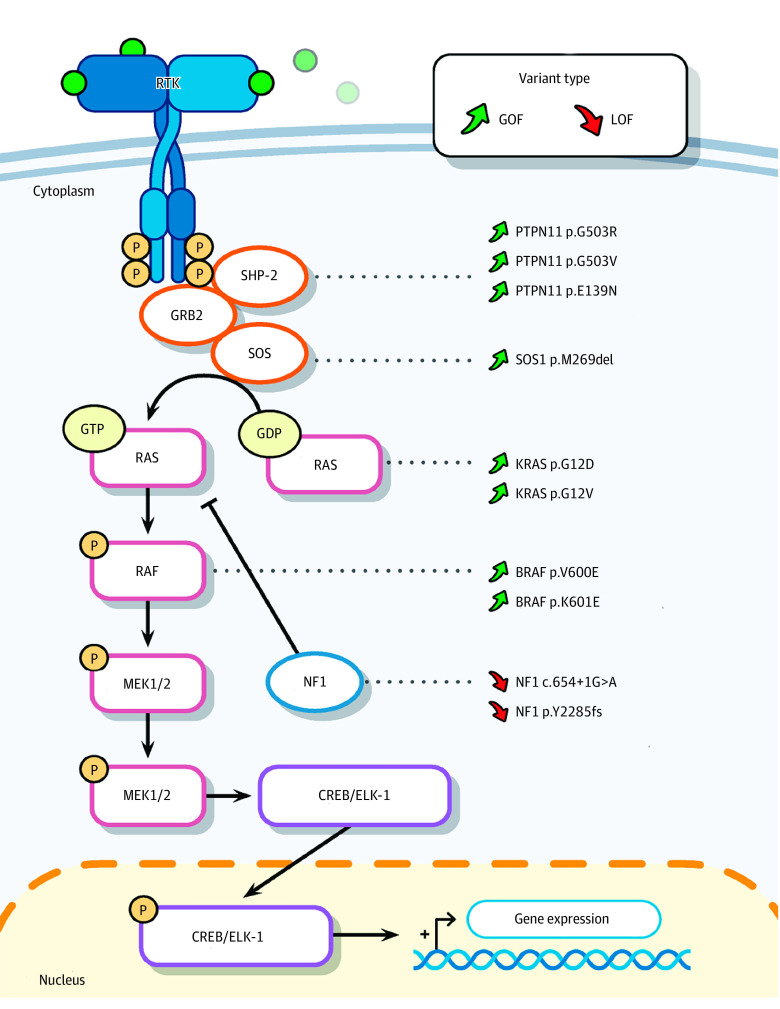
Somatic Variants Activating Ras/Raf/Mitogen-Activated Protein Kinase (MAPK) Pathway Genes in Mesial Temporal Lobe Epilepsy Simplified diagram of the Ras/Raf/MAPK signaling pathway, the pathogenic variants discovered in the mesial temporal lobe epilepsy cohort, and their corresponding proteins. GDP indicates guanosine diphosphate; GOF, gain of function; GTP, guanosine triphosphate; LOF, loss of function; RTK, receptor tyrosine kinase.

All but 1 variant (*SF3B1* p.K700E) in the Table are present in Ras/Raf/mitogen-activated protein kinase (MAPK) pathway genes (Figure 1A). Notably, all the variants are predicted to increase Ras/Raf/MAPK signaling via gain of function of pathway activators (*PTPN11*, *KRAS*, *SOS1*, or *BRAF*) or loss of function of a pathway repressor (*NF1*). To determine whether patients with activating Ras/Raf/MAPK variants have unique clinical characteristics, we independently reviewed all available clinical data, magnetic resonance images, and histopathology slides for this subgroup. All variant-positive patients were seizure-free for more than 2 years after surgery (Engel class IA or International League Against Epilepsy class 1), with significantly increased likelihood of Engel class IA outcome compared with the rest of the cohort (eFigure 2 in Supplement 1). All the patients with pathogenic Ras/Raf/MAPK variants had evidence of MTS; in 5 patients, that was the only major imaging and histopathologic finding (eFigure 3A-P in Supplement 1) while the remaining half exhibited additional findings consistent with FCDs or LEATs (eFigure 4 in Supplement 1). To further rule out the possibility of radiographically and histopathologically undetected LEATs, we performed additional CD34 staining—a diagnostic marker for LEATs^24^—in 4 samples with MTS-only pathology for which tissue was available. We did not observe higher than background-level CD34 staining (eFigure 3M-P in Supplement 1), further confirming that Ras/Raf/MAPK variants can be present at low VAFs in the affected temporal lobe in the absence of a glioneuronal tumor detected radiographically or histopathologically.

### Enrichment of Activating Ras/Raf/MAPK Pathway Variants in MTLE

**Figure 2.**
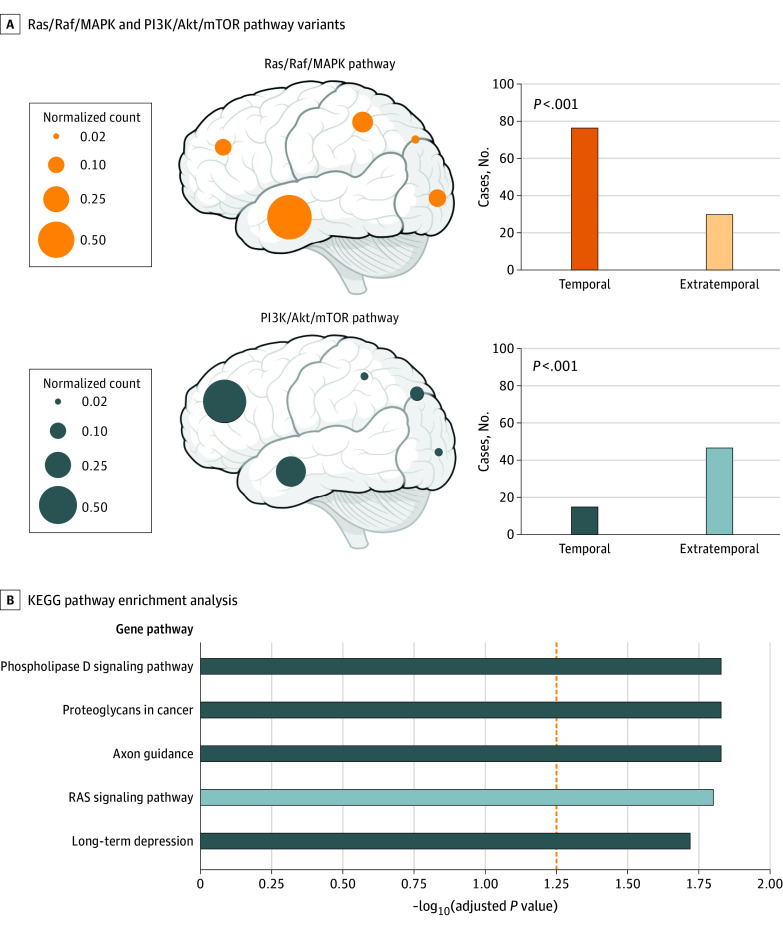
Enrichment of Ras/Raf/Mitogen-Activated Protein Kinase (MAPK) Pathway Variants in the Temporal Lobe and in Mesial Temporal Lobe Epilepsy (MTLE) A, Retrospective review of Ras/Raf/MAPK pathway variants and PI3K/Akt/mammalian target of rapamycin (mTOR) pathway variants in the lesional focal epilepsy literature. Circle diameters represent normalized case counts in each brain region and the associated bar plots on the right depict the absolute number of cases. B, Kyoto Encyclopedia of Genes and Genomes (KEGG) pathway enrichment analysis on the pathogenicity-enriched variants in the MTLE cohort (*P* < .001 and adjusted *P* < .05 after correction for multiple hypotheses testing). The dashed orange line represents the adjusted *P* value of .05.

While the contribution of somatic variants to MTLE has not been previously described, to our knowledge, most FCD-associated somatic variants reported to date are in PI3K/Akt/mTOR pathway genes,^15^ whereas LEAT-associated variants often involve the Ras/Raf/MAPK pathway.^24^ To further investigate the association between somatic genotype and brain regional specificity, we performed a focused retrospective review of the FCD and LEAT literature. Our analysis demonstrated a significant predilection of somatic Ras/Raf/MAPK variants for the temporal lobe, while PI3K/Akt/mTOR variants showed an extratemporal predominance (Figure 2A). Given that the hippocampus is the primary affected region in MTLE, our findings lend further support to the hypothesis that somatic variants in the Ras/Raf/MAPK pathway arising specifically in the temporal lobe confer risk for focal epilepsy.

Since our initial detection of pathogenic somatic variants relied on prior reporting of a specific variant in ClinVar, we next performed pathway enrichment analysis on all the filtered variants from our call set that were predicted to be pathogenic (eTables 2 and 3 in Supplement 2). Consistent with our initial observation, for somatic variants in the MTLE cohort, the Ras/Raf/MAPK signaling pathway was among the most highly enriched pathways (Figure 2B). Enrichment in this pathway was not seen in the control samples. Furthermore, we showed that pathogenicity-enriched variants in another curated Ras/Raf/MAPK gene set (eTable 3 in Supplement 1) are overrepresented in the MTLE cohort and not the control samples. Notably, we did not see an overrepresentation of PI3K/Akt/mTOR genes (eTable 3 in Supplement 1) in the MTLE cohort, further supporting the notion that focal epilepsies in the temporal lobe share a genetic etiology distinct from focal extratemporal epilepsies.

### Mechanism of Ras/Raf/MAPK Overactivation in MTLE-Associated *PTPN11* Somatic Variants

**Figure 3.**
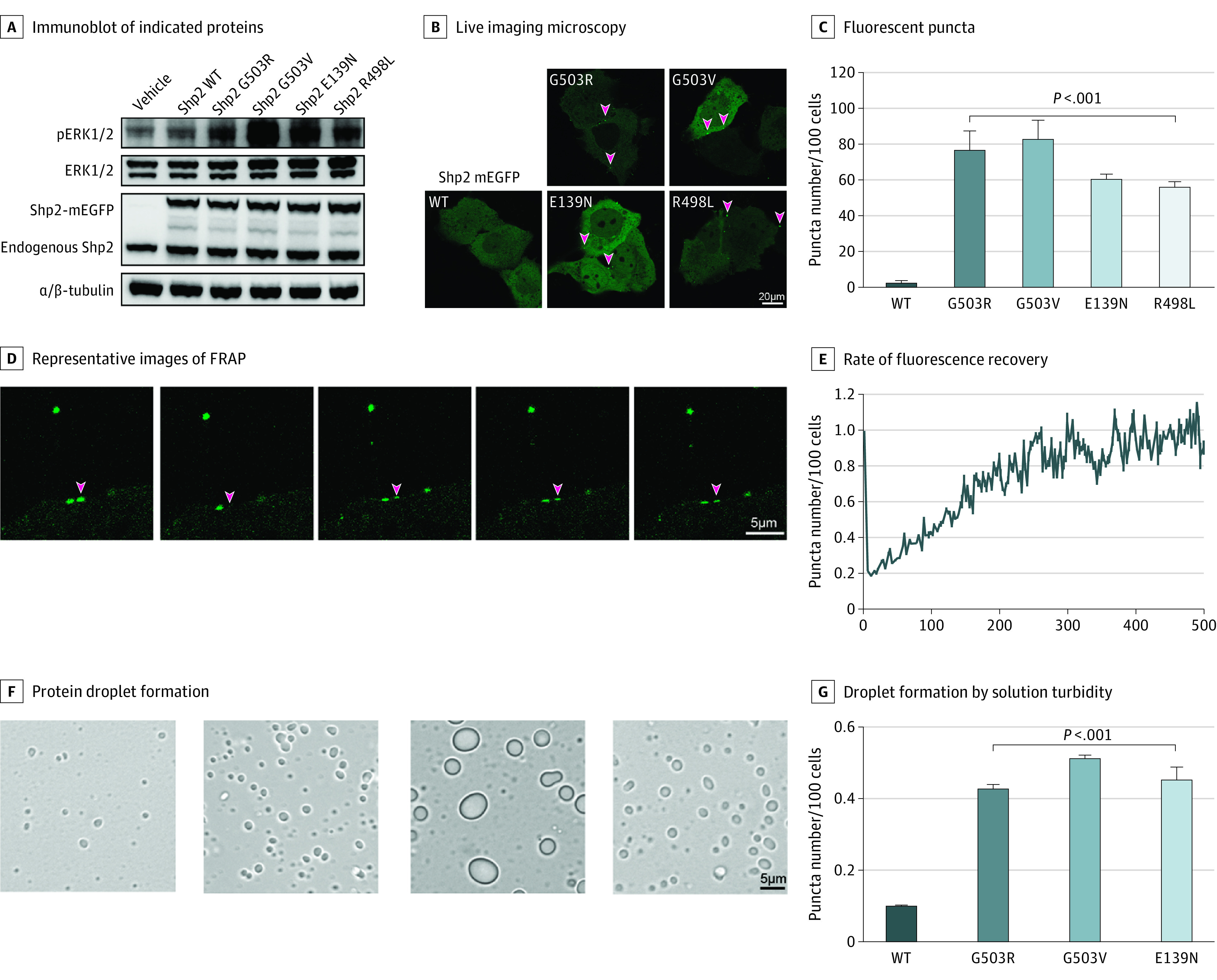
Mechanisms of Ras/Raf/Mitogen-Activated Protein Kinase (MAPK) Overactivity in Mesial Temporal Lobe Epilepsy–Associated Variant Shp2 Proteins A, Immunoblot of the indicated proteins in HEK293T cells transiently expressing wild-type (WT) and variant Shp2. B, Representative pictures from live imaging microscopy of monomeric enhanced green fluorescent protein (mEGFP)–labeled WT and variant Shp2 proteins, transiently expressed in KYSE520 cells. C, The fluorescent puncta (marked by arrowheads in panel B) are quantified. D, Representative images of fluorescence recovery after photobleaching (FRAP) using the transiently expressed mEGFP-labeled G503R Shp2 protein in KYSE520 cells. E, The rate of fluorescence recovery in panel D is quantified. F, Representative images of 8-μM WT and variant Shp2 protein droplet formation in the presence of 10% (w/v) PEG3350 in vitro. G, Droplet formation is quantified by solution turbidity of OD600.

Germline *PTPN11* variants, which are known causes of Noonan syndrome and related disorders, appear to enhance protein tyrosine phosphatase enzymatic activity through an acquired capability of LLPS of the variant Shp2 protein encoded by *PTPN11* variants.^26^ Since this gene, to our knowledge, has not been previously associated with focal epilepsy, we evaluated the functional impact of MTLE-associated *PTPN11* variants by transiently expressing the human wild type (wild-type Shp2) and variant (variant Shp2) constructs in HEK293T cells and then performing immunoblotting to assess the degree of Erk1/2 (a downstream effector of the Ras/Raf/MAPK pathway) phosphorylation. Compared with cells expressing the wild-type Shp2 protein, we detected increased phosphorylated Erk1/2 (pErk1/2) in cells expressing variant Shp2 proteins (Figure 3A), indicating increased phosphatase activity of all the Shp2 variants, consistent with constitutive activation.

To determine the mechanism through which MTLE-associated *PTPN11* variants enhance phosphatase activity, we tested whether these variants also undergo LLPS. We transiently expressed monomeric enhanced green fluorescent protein (mEGFP)–labeled Shp2 proteins (wild type, G503R, G503V, E139N, and R498L) in KYSE520 cells. Remarkably, the Shp2 variants formed discrete puncta in cells, whereas the wild-type protein was dispersed throughout the cell (Figure 3B and C). Fluorescence recovery after photobleaching experiments showed that mEGFP-labeled G503R Shp2 puncta recovered within minutes after photobleaching (Figure 3D and E), indicating that the variant Shp2 proteins formed condensates that exhibited dynamic liquidlike behavior. To explore whether variant Shp2 proteins could also undergo LLPS in vitro, we expressed and purified recombinant Shp2 variant and wild-type proteins. The in vitro droplet formation assay^26^ showed that the variant Shp2 proteins formed more and larger droplets compared with the wild-type protein in the presence of 10% (w/v) PEG3350, which was quantified using solution turbidity of OD600 (Figure 3F and G). These findings indicate that the MTLE-associated variant Shp2 proteins undergo LLPS in cells and in vitro through a dominant gain-of-function mechanism that activates downstream Ras/Raf/MAPK signaling.

### Ras/Raf/MAPK Overactivation in Affected Human MTLE Surgical Tissue

**Figure 4.**
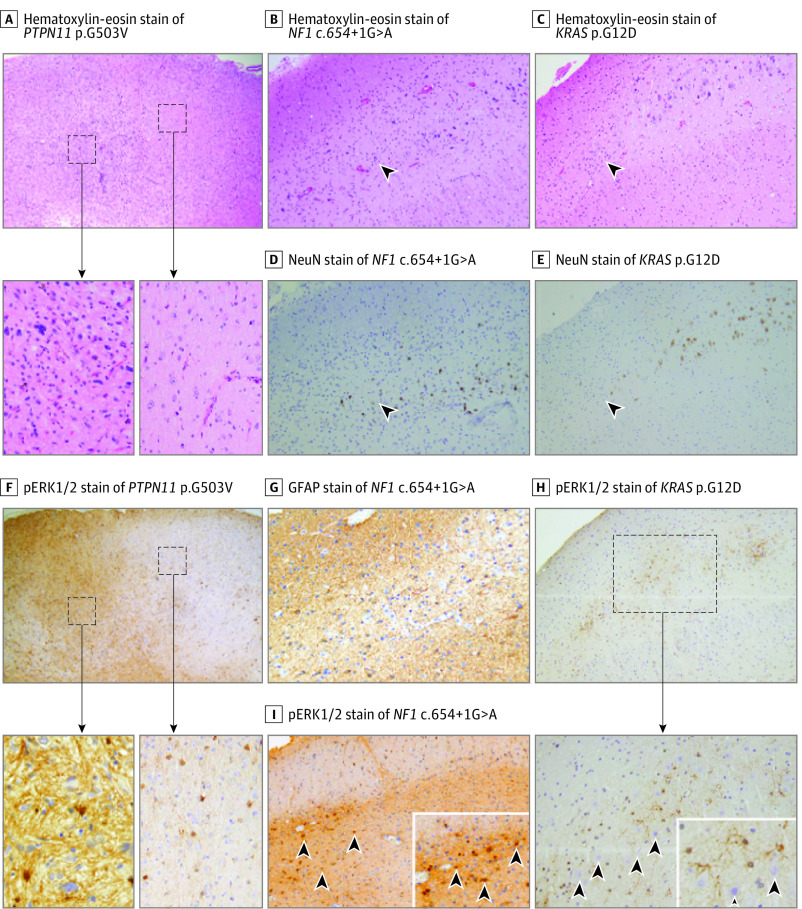
Evidence of Erk1/2 Overactivation in Mesial Temporal Lobe Epilepsy Tissue Harboring Ras/Raf/Mitogen-Activated Protein Kinase Variants Each column shows histopathologic images from 1 sample. A and F, A low-grade epilepsy–associated tumor. Original magnification ×40 for image and ×400 for inset. A, Hematoxylin-eosin staining with areas of hypercellularity corresponding to the tumor (inset 1) intermixed with normal brain tissue (inset 2). F, pErk1/2 staining of the corresponding regions is seen in insets 1 and 2. B to E and G, Representative mesial temporal sclerosis–only samples with neuronal dropout in the cornu ammonis region of the hippocampus (panels B to E) but uniform distribution of glial cells across the same region (panel G). The black arrowheads demarcate cornu ammonis neuronal loss. Original magnification ×200. H, Original magnification ×40 for image and ×200 for inset. I, Original magnification ×200. H and I, Erk1/2 phosphorylation (pErk1/2) colocalizes to regions with highest degree of neuronal loss and sclerosis. The arrowheads point to cells with increased pErk1/2 staining, mostly represented by apparent glia (panel I, inset). The black arrowheads point to neurons that have relatively low pErk1/2 staining (panel H, inset).

Given the enrichment of activating Ras/Raf/MAPK somatic variants in MTLE, the variant-positive cells could play a key and potentially causal role in hippocampal epileptogenesis. In the absence of allele-specific antibodies for most of the variants found in our cohort, we used immunohistochemical staining for pErk1/2 (a proxy for Ras/Raf/MAPK pathway overactivation^27^) to determine the identity and localization of variant-positive cells. In brain tissue from the patient with LEAT associated with *PTPN11* p.G503V, we observed substantial correlation between tumor density (Figure 4A) and the degree of pErk1/2 staining (Figure 4F), supporting the validity of this strategy in localizing variant-positive cells. Using this approach in 2 patients with *NF1* c.654 + 1G>A and *KRAS* p.G12D variants and MTS-only pathology, we observed that hippocampal subregions with the greatest neuronal loss showed the highest density of pErk1/2 staining (Figure 4B to E, H, and I). Most cells with intense pErk1/2 staining in areas of neuronal loss showed glial morphology (Figure 4G to I), which could not be explained by the differences in cell type–specific gene expression in the adult human hippocampus (eFigure 5 in Supplement 1).

## Discussion

We identified pathogenic somatic variants enriched in the hippocampus, where seizures in MTLE typically originate, in 11 patients with drug-resistant MTLE, with none found in the neurotypical controls. Strikingly, all but 1 of these variants were predicted to activate Ras/Raf/MAPK signaling, providing strong evidence that they may contribute to MTLE risk, analogous to the role of somatic PI3K/Akt/mTOR variants in FCD. The finding that even MTLE cases without evidence of dysplasia or neoplasia carry pathogenic somatic variants in the Ras/Raf/MAPK pathway could significantly alter our understanding of, and the therapeutic options for, this most common indication for epilepsy surgery.

The regional specificity of epilepsy-associated Ras/Raf/MAPK pathway variants in the temporal lobe, vs PI3K/Akt/mTOR pathway variants in the extratemporal cortex, recalls cancers where common driver variations are characteristic to particular cell and tissue types.^19^ Hence, the regional genetic specificity of focal epilepsies may represent the major differences in cellular architecture and proliferative properties between different brain regions. The higher VAFs of Ras/Raf/MAPK variants in the hippocampus compared with the lateral temporal neocortex parallels the pattern of ongoing neurogliogenesis, which continues after birth only in the dentate gyrus,^28^ and may contribute to this enrichment. Febrile seizures and head trauma, well-established risk factors for MTLE,^3,4^ stimulate this dentate gyrus proliferation in animal models.^29,30^ A similar process in humans could create a proliferative or survival advantage for cells harboring activating Ras/Raf/MAPK pathway variants, as has been described in cancer,^31^ and could provide a potential mechanism for how these risk factors may contribute to MTLE risk.

We also observed a significant association between the lower VAF of pathogenic variants and the absence of dysplasia or neoplasia on imaging and histopathology. This finding implies that patients with MTS-only pathology may have acquired their variants at later developmental stages^15^ and therefore have fewer variant-positive cells. Patients with MTS with LEATs or FCD pathology, in contrast, may have acquired their variants earlier, resulting in higher fractions of variant-positive cells and diagnosis of drug-resistant epilepsy at a younger age, as illustrated by patients 7 and 10 (Table), who underwent epilepsy surgery as infants. Most patients in our cohort are adults, and because of limitations of retrospective access to clinical information, we were unable to test the association between the age of onset or epilepsy duration with VAF. In addition to VAF, other factors may contribute to the histopathology associated with Ras/Ras/MAPK variants. For example, patients 1 and 2 both have *PTPN11* variants with similar VAFs that change the same amino acid, but a greater degree of Ras-MAPK activation by *PTPN11* p.G503V may account for MTS with LEATs or MTS with FCD pathology as opposed to MTS-only pathology with p.G503R. The cellular lineage of variant-positive cells is likely an independent predictor of pathology as well.^32^ Consequently, somatic Ras/Raf/MAPK pathway variants may give rise to a spectrum of temporal lobe lesions depending on the developmental time point at which they were acquired, their molecular mechanisms, and the cell types of variant-positive cells, all associated with drug-resistant MTLE.

To our knowledge, the mechanism through which somatic Ras/Raf/MAPK variants contribute to epileptogenesis is yet to be determined, although our functional data support the role of Ras/Raf/MAPK overactivation in this process. While all variant-positive patients exhibited histopathologic evidence of MTS, we did not identify pathogenic somatic variants in most patients with MTS, and very few patients with non-MTS histopathology were enrolled in the study; therefore, the exact association between MTS and pathogenic somatic variants remains unclear based on our data. The precise cell type–specific effects of somatic Ras/Raf/MAPK pathway variants in MTLE are likely quite complicated and may evolve over time, given that these variants can cause different phenotypes in distinct cell types, contexts, and brain regions.^33,34^ Our human genetic findings provide the impetus to explore these hypotheses in future work with animal models.

Our study highlights the importance of incorporating molecular testing into the MTLE diagnostic algorithm. Although it is not known yet whether MTLE-associated somatic variants can be detected less invasively, perhaps presurgical evaluation of cell-free DNA in cerebrospinal fluid^35,36^ or genomic DNA in stereoelectroencephalography electrodes^37^ could guide further management steps, such as surgical approaches vs genotype-driven therapeutics. For example, in patients with left MTLE who may be at risk of significant verbal memory or language decline with an extensive resection, or in patients who do not receive surgery because of patient/physician perception^38^ or limited resources,^39^ a genotype-driven pharmacological approach may provide an additional treatment option. Since many targeted treatments for Ras/Raf/MAPK activation are already in various stages of clinical testing in cancers,^40^ our findings offer the potential to leverage some of these agents to develop the first generation of targeted therapies for molecularly characterized MTLE.

### Limitations

This study has limitations. Despite the high sequencing depth, somatic variant detection was mostly limited to VAFs greater than 1% due to technical factors. Since cell loss is a prominent feature of MTS, pathogenic variants in the hippocampus may be present in VAFs less than 1%, suggesting that the burden of pathogenic somatic variants in MTLE could be much higher than what is reported here.

## Conclusions

Hippocampal somatic variants, particularly those activating Ras/Raf/MAPK signaling, may contribute to the pathogenesis of sporadic, drug-resistant MTLE. These findings may provide a novel genetic mechanism and highlight new therapeutic targets for this common indication for epilepsy surgery.

## Notes

Supplement 1.
eMethods.
**eTable 1.** Summary of Demographic and Clinical Characteristics of Patients With MTLE and Neurotypical Controls **eTable 2.** Demographic Information, Cause of Death, Tissue Availability, Source, and Sequencing Strategy for Neurotypical Controls **eTable 3.** Curated Gene Sets for Overrepresentation Analysis **eFigure 1.** Mean Sequencing Depth for MTLE and Neurotypical Control Samples **eFigure 2.** Two-Year Epilepsy Surgery Outcome **eFigure 3.** MTLE Cases With Activating Ras/Raf/MAPK Variants and MTS-Only Pathology **eFigure 4.** MTLE Cases With Activating Ras/Raf/MAPK Variants and MTS+ Pathology **eFigure 5.** Expression of Ras/Raf/MAPK Genes With MTLE-Associated Somatic Variants in the Adult Human Brain
eReferences.

Supplement 2. **eTable 1.** Demographic Information, Clinical Characteristics, Tissue Availability, Recruitment Site, 2-Year Surgical Outcome, and Sequencing Strategy for Patients With MTLE **eTable 2.** List of All the Filtered Variants Predicted to be Pathogenic In Silico Based on REVEL, EVE, and SpliceAI scores in MTLE Call Set **eTable 3.** List of All the Filtered Variants Predicted to be Pathogenic In Silico Based on REVEL, EVE, and SpliceAI scores in Control Call Set

Supplement 3. Data Sharing Statement
