# Supplementary material for: Contribution of Somatic Ras/Raf/Mitogen-Activated Protein Kinase Variants in the Hippocampus in Drug-Resistant Mesial Temporal Lobe Epilepsy
Source: JAMA Neurol. 2023 May 1;80(6):578–87. doi: 10.1001/jamaneurol.2023.0473 (PMC10152377; doi:10.1001/jamaneurol.2023.0473)
Supplement: Supplement 3. — Data Sharing Statement [file jamaneurol-e230473-s003.pdf]

Data Sharing Statement

Khoshkhoo. Contribution of Somatic Ras/Raf/Mitogen-Activated Protein Kinase Variants in the Hippocampus in Drug-Resistant Mesial Temporal Lobe Epilepsy. *JAMA Neurol*. Published May 01, 2023. doi:10.1001/jamaneurol.2023.0473

Data
Data available: Yes
Data types: Data (not involving human participants)
How to access data: All the sequencing data generated for this study will be deposited in the database of Genotypes and Phenotypes (dbGaP) and may be accessed through the following accession numbers: phs000492.v4.p2 and phs002128.v1.
When available: With publication

Supporting Documents
Document types: None

Additional Information
Who can access the data: Researchers whose use of the data is consistent with the consent document data use limitations.
Types of analyses: Research studies of epilepsy and neurological diseases.
Mechanisms of data availability: without investigator support with a signed data access agreement.
